# Understanding MOG antibody-associated disease in Brazil

**DOI:** 10.1055/s-0043-1777298

**Published:** 2023-11-30

**Authors:** Dagoberto Callegaro, Guilherme Diogo Silva

**Affiliations:** 1 Universidade de São Paulo, Faculdade de Medicina, Hospital das Clínicas, São Paulo SP, Brazil.

In this issue of *Arquivos de Neuropsiquiatria* , Messias and colleagues report on a series of 41 patients with MOG antibody-associated disease (MOGAD) in a Brazilian tertiary center in São Paulo. 1 MOGAD presents a broad spectrum of clinical presentations and a detailed phenotype description is important to prevent misdiagnosis with other demyelinating diseases, such as multiple sclerosis or neuromyelitis optica spectrum disorder. Moreover, the extensive racial mingling and the scarcity of reports on MOGAD in Latin-Americans motivate studies describing the clinical and paraclinical features of MOGAD in Brazil.

The first interesting finding of the study was that Brazilian MOGAD patients presented with phenotypes similar to what was reported from other regions. 2 Therefore, Brazilian neurologists should understand the classical phenotypes of MOGAD. Optic neuritis emerged as the most common phenotype, identified in over 80% of adult-onset MOGAD cases. A previous study of 77 patients with first-ever optic neuritis in São Paulo showed the clinical features of MOGAD and multiple sclerosis frequently overlap as unilateral, subacute, painful, visual loss with afferent pupillary defect. 3 However, unselected testing for anti-MOG IgG antibodies could lead to false positive results, especially considering that adult-onset MOGAD is over 30 times rarer compared with multiple sclerosis in São Paulo. 4 In the research led by Messias and colleagues, atypical characteristics of multiple sclerosis associated optic neuritis were frequently observed in MOGAD patients. These characteristics included abnormal fundoscopy, longitudinally extensive involvement of the optic nerve, and perineuritis, documented in 60%, 75%, and 83% of cases, respectively. Not only a higher frequency of disc edema, but also maculopathy have been described in patients with MOGAD. 5

Ten percent of MOGAD patients presented with transverse myelitis. However, compared with patients with AQP4 antibodies, patients with MOG antibodies have a higher frequency of spinal cord lesions distributed in the lower portion of the spinal cord. 6 A third of the reported cases of myelitis presented conus involvement. Another interesting finding of this study was that conus involvement was particularly common when associated with another phenotype of MOGAD, the acute disseminated encephalomyelitis (ADEM).

ADEM represents the predominant encephalitic manifestation of MOGAD. Yet, the presence of cortical encephalitis in two patients underscores the importance of considering anti-MOG antibodies when diagnosing autoimmune encephalitis, even in the absence of radiological indicators of central nervous system demyelinating disease. 7 This consideration becomes especially pertinent in pediatric cases, given that six of the seven encephalitis cases were observed in children.

Messias and colleagues also discussed the relationship between MOGAD and vaccines. Approximately one-third of the subjects had experienced infections or had vaccinations prior to the onset of MOGAD. Moreover, the study presents the first report of MOGAD associated with Sinovac-CoronaVac vaccine technology. However, large population studies failed to establish any causal association between vaccines and risk of developing central nervous system demyelinating diseases. 8 Further research is required to improve our understanding of this topic.

The study showed the current treatment strategies used in MOGAD in Brazil. Azathioprine was the most commonly used first-line immunotherapy, aligning with findings from another Brazilian research. 9 Although there is a trend in the field of Neuroimmunology for early high-efficacy immunotherapy in multiple sclerosis, 10 the benefit of this strategy in MOGAD patients is not clear. Moreover, the study reinforced that the prognosis of MOGAD is frequently favorable, with half of the reported MOGAD patients presenting a monophasic course and exhibiting low residual disability at the last follow-up.

Hence, through a detailed phenotypic characterization, Messias and colleagues reinforce the current understanding that the anti-MOG antibody is not a predictor of progression to multiple sclerosis or a marker for neuromyelitis optica in patients negative for the anti-AQP4 antibody, but rather an indicator of a distinct disease entity. 2
